# Changes in monocyte subsets in volunteers who received an oral wild-type *Salmonella* Typhi challenge and reached typhoid diagnosis criteria

**DOI:** 10.3389/fimmu.2024.1454857

**Published:** 2024-08-27

**Authors:** Franklin R. Toapanta, Jingping Hu, Kari Ann Shirey, Paula J. Bernal, Myron M. Levine, Thomas C. Darton, Claire S. Waddington, Andrew J. Pollard, Marcelo B. Sztein

**Affiliations:** ^1^ Department of Medicine and Center for Vaccine Development and Global Health, University of Maryland School of Medicine, Baltimore, MD, United States; ^2^ Department of Microbiology and Immunology, University of Maryland School of Medicine, Baltimore, MD, United States; ^3^ Department of Pediatrics and Center for Vaccine Development and Global Health, University of Maryland School of Medicine, Baltimore, MD, United States; ^4^ Oxford Vaccine Group, Department of Paediatrics, University of Oxford and the National Institute for Health and Care Research (NIHR) Oxford Biomedical Research Centre, Oxford, United Kingdom

**Keywords:** S. Typhi, human oral challenge, CHIM, classical monocytes, intermediate monocytes, non-classical monocytes

## Abstract

An oral Controlled Human Infection Model (CHIM) with wild-type *S*. Typhi was re-established allowing us to explore the development of immunity. In this model, ~55% of volunteers who received the challenge reached typhoid diagnosis criteria (TD), while ~45% did not (NoTD). Intestinal macrophages are one of the first lines of defense against enteric pathogens. Most organs have self-renewing macrophages derived from tissue-resident progenitor cells seeded during the embryonic stage; however, the gut lacks these progenitors, and all intestinal macrophages are derived from circulating monocytes. After infecting gut-associated lymphoid tissues underlying microfold (M) cells, *S*. Typhi causes a primary bacteremia seeding organs of the reticuloendothelial system. Following days of incubation, a second bacteremia and clinical disease ensue. *S*. Typhi likely interacts with circulating monocytes or their progenitors in the bone marrow. We assessed changes in circulating monocytes after CHIM. The timepoints studied included 0 hours (pre-challenge) and days 1, 2, 4, 7, 9, 14, 21 and 28 after challenge. TD participants provided extra samples at the time of typhoid diagnosis, and 48-96 hours later (referred as ToD). We report changes in Classical Monocytes -CM-, Intermediate Monocytes -IM- and Non-classical Monocytes -NCM-. Changes in monocyte activation markers were identified only in TD participants and during ToD. CM and IM upregulated molecules related to interaction with bacterial antigens (TLR4, TLR5, CD36 and CD206). Of importance, CM and IM showed enhanced binding of *S*. Typhi. Upregulation of inflammatory molecules like TNF-α were detected, but mechanisms involved in limiting inflammation were also activated (CD163 and CD354 downregulation). CM upregulated molecules to interact/modulate cells of the adaptive immunity, including T cells (HLA-DR, CD274 and CD86) and B cells (CD257). Both CM and IM showed potential to migrate to the gut as integrin α4β7 was upregulated. Unsupervised analysis revealed 7 dynamic cell clusters. Five of these belonged to CM showing that this is the main population activated during ToD. Overall, we provide new insights into the changes that diverse circulating monocyte subsets undergo after typhoid diagnosis, which might be important to control this disease since these cells will ultimately become intestinal macrophages once they reach the gut.

## Introduction

*Salmonella enterica* serovar Typhi (*S*. Typhi) is a human restricted pathogen and the agent responsible for typhoid fever. This pathogen continues to be a major public health problem, particularly in developing countries, where young children are disproportionately affected (1–5). The innate immune system is one of the first lines of defense against invading pathogens. Among innate cells, circulating monocytes, derived from bone marrow precursors, show great plasticity and can differentiate into various cell types (e.g., macrophages and dendritic cells -DC-) once they reach their homing organs and are exposed to the appropriate cytokine environment (6–11). During steady state, tissue-resident macrophages from most organs (e.g., brain, liver, lungs) are renewed by tissue-resident progenitor cells that are seeded before birth (12, 13). During acute infections, the pool of macrophages in these tissues increase due to the influx of circulating monocytes that differentiate into macrophages (monocyte-derived macrophages) (14–19). Importantly, the gut lacks tissue-resident macrophage-progenitors; therefore, all macrophages in the gut are monocyte-derived, even those present during steady state (20–24). Animal studies have shown the importance of local macrophages in controlling bacterial growth (25–27); therefore, it is important to determine whether circulating monocytes, which will contribute to the pool of tissue macrophages are affected by *S*. Typhi infection.

*S.* Typhi is transmitted by the fecal oral route (3, 28). Following ingestion, the bacteria enter the host via the intestinal mucosa. After infecting gut-associated lymphoid tissues (GALT) underlying microfold (M) cells, S. Typhi uses the GALT lymphatic vessels to reach the great veins (blood) and disseminate systemically seeding organs of the reticuloendothelial system (e.g., liver, spleen, bone marrow). This primary bacteremia (silent) occurs shortly after ingestion of the bacterial inoculum and individuals do not show any clinical symptoms. After a moderately long incubation time (~8-14 days), some individuals will develop a febrile illness. This is accompanied by a low-level bacteremia (1-10 CFU/ml of blood) (29) (secondary bacteremia) (30). The advent of optimized culture-PCR assays has allowed detection of the primary bacteriemia in some volunteers in challenge studies with wild-type *S*. Typhi (30, 31). Classic blood culture tests usually detect *S*. Typhi during the secondary bacteremia (29, 32). *S.* Typhi or bacterial products/metabolites likely encounter bone marrow monocyte precursors and/or blood monocytes during the primary and/or secondary bacteremia. Whether these encounters alter the phenotype of the monocytes remains unknown. This interaction can have important consequences for the host since all gut macrophages, as well as a portion of macrophages in other tissues, are derived from monocytes in circulation.

In humans, the blood monocyte compartment is composed of various subsets identified by the distinct expression of CD14 and CD16. These subsets include Classical Monocytes (CM; CD14+ CD16-), Intermediate Monocytes (IM; CD14+ CD16+) and Non-classical Monocytes (NCM; CD14dim CD16+). CM make up ∼85% of the circulating monocyte pool, whereas the remaining ∼15% consists of IM and NCM (33, 34). Mouse and human studies showed that IM and NCM represent diverse stages in the developmental sequence of monocytes (12, 35–38). CM have a short lifespan in blood (~1-2 days) and more than 95% of these cells are rapidly recruited to tissues to support tissue-resident macrophages (38). CMs that remain in circulation transform into IM, which have a lifespan of ~4 days. IM have similar functional characteristics than CM. Circulating IM eventually become NCM, which represent the final stage in the development of circulating monocytes and are considered luminal blood macrophages (39). These cells have a lifespan of ~7-8 days (38) and have been proposed to act as custodians of the vasculature by patrolling endothelial cell integrity in an LFA-1-dependent fashion (40).

In recent years, studies have tried to understand the role that the diverse monocyte subsets play in health and disease. Reports have shown changes in the frequencies of these subsets during bacterial and viral infections; for example, sepsis patients showed an expansion of the IM and NCM subsets (41–43). Interestingly, during steady state, IM have the highest expression of surface markers for antigen processing and presentation, such as HLA-DR (34, 44). Expression of this marker is significantly reduced in sepsis patients, when compared with healthy controls (42, 43, 45). The degree of recovery in surface expression of this marker is associated with mortality as survivors show an increase HLA-DR expression while non-survivors fail to recover expression of this marker (46–48). Of note, HLA-DR expression changes in sepsis were observed in all monocyte subsets. Whether similar changes are induced by *S*. Typhi infection during primary or secondary bacteremia remain unknown. In this study, we used specimens from a controlled human infection model of wild-type (wt) *S.* Typhi that was reestablished by the Oxford Vaccine Group at the University of Oxford. In this outpatient model, challenge with 10^3^ CFU of *S.* Typhi (Quailes strain) resulted in ~55% of participants reaching typhoid endpoints (referred herein as typhoid diagnosis -TD-) (49). Specimens from volunteers who reached TD, or not, (TD and NoTD, respectively) were used to study the various monocyte subsets. Importantly, in a previous study from our group, we started characterizing some aspects of the monocyte response to wt *S*. Typhi infection. We showed that monocytes of TD volunteers during time of disease had an activated phenotype (e.g., increase expression of CD38 and CD40), upregulated molecules involved in migration to GALT (e.g., integrin α4β7) and had enhanced ability to bind *S*. Typhi (50). In the current study, we extended our studies and evaluated in depth the characteristics of various circulating monocytes subsets. In these subsets we assessed expression of markers involved in the interaction with bacteria (TLR-4, TLR-5, TREM-1, CD36, CD163) as well as markers involved in antigen presentation and/or interaction with cells of the adaptive immune response (HLA-DR, CD86, CD274, CD257). Moreover, we assessed the ability of the monocyte subsets to interact with *S*. Typhi and migrate to the gut as determined by expression of integrin α4β7. The results showed that a multiplicity of changes in the monocyte subsets are elicited following *S*. Typhi infection.

## Materials and methods

### Participants, clinical trial description and ethics statement

Samples used in this study were collected as part of a clinical study to establish a human model of typhoid fever (Oxford Vaccine Group, University of Oxford, United Kingdom). The details of the clinical outcomes have already been published (49). In brief, healthy adults (18-60 years-old) with no previous history of typhoid vaccination or residence (>6 months) in endemic areas were enrolled. Volunteers ingested a *S.* Typhi (Quailes strain) inoculum (10^3^ or 10^4^ CFU) as previously described (49). Typhoid diagnosis included meeting clinical (temperature ≥38°C sustained for ≥ 12 hours) and/or microbiological (blood culture confirmed *S*. Typhi bacteremia) endpoints. Participants were monitored daily for at least 14 days. The duration and severity of all solicited and unsolicited symptoms experienced were recorded daily as well as oral temperature readings (twice per day). All participants received antibiotic treatment (ciprofloxacin, 500 mg twice daily, 14 days) at typhoid diagnosis, or at day 14 post challenge (or if felt clinically necessary prior to day 14 or typhoid diagnosis). Subsequent visits were performed at days 21 and 28 after challenge. In the current report we describe the results from a subset of volunteers challenged with 10^3^ CFU of *S*. Typhi. In this subset (n=10), 5 volunteers were diagnosed with typhoid disease (TD) and 5 were not (NoTD). Specimens for this study were selected based on the availability of critical time points to perform a comprehensive evaluation of monocytes subsets. This clinical study was performed by the Oxford Vaccine Group at the Centre for Clinical Vaccinology and Tropical Medicine of the University of Oxford (UK) (49). All volunteers provided a written informed consent at the time of enrollment and the procedures were approved by the Oxfordshire Research Ethics Committee A (10/H0604/53). This clinical study was registered on the UK Clinical Research Network (identifier UKCRN ID 9297).

### Isolation of peripheral blood mononuclear cells (PBMC)

Blood specimens were collected from *S.* Typhi challenged volunteers and PBMC isolated by density gradient centrifugation and cryopreserved as previously described (49). Blood specimens were collected before challenge and at various time points thereafter. The evaluated time points differed slightly between TD and NoTD volunteers. These included 0 hours (pre-challenge) and days 1 (24h), 2, 4, 7, 9, 14, 21 and 28 after challenge in all subjects. In TD volunteers extra samples were collected at the time of typhoid diagnosis (9-14 days after challenge with 10^3^ CFU of wt *S.* Typhi (49),) as well as 48 and 96 hours later.

### Cell surface staining for flow-cytometry

Cryopreserved PBMC were thawed and after resting for 2-3 hours stained for flow cytometry analyses. In specimens in which TNF-α was assessed, Golgi apparatus inhibitors (Brefeldin A and Monensin) were added immediately after thawing the cells. Following 3 hour of incubation (37°C, 5% CO_2_) the cells were stained for flow cytometry. PBMC were cultured in complete media [RMPI (Gibco, NY, USA) supplemented with 10% fetal bovine serum (FBS) (Gemini Bioproducts, West Sacramento, CA), 2 mM L-glutamine (Gibco, Grand Island, NY, USA), 1x non-essential amino acids (Gibco, Grand Island, NY, USA), 10 mM HEPES (Gibco, Grand Island, NY, USA), 2.5 mM Sodium pyruvate, (Lonza, Walkersville, MD, USA), 100 U/ml Penicillin, 100 ug/ml streptomycin (Sigma-Aldrich, St. Louis, MO, USA), 50 μg/ml Gentamicin (Gibco, Grand Island, NY, USA)]. Cells were stained for flow cytometry in V-shaped 96-well plates using methods previously described (50, 51). Briefly, 1x10^6^ cells were plated and stained for viability (20 min on ice) using 100 μl of Alexa Fluor 700-succinimidyl ester dye (0.4 μg/ml) (Invitrogen, USA). After 2 washes with flow cytometry staining (FC) buffer (4% FCS, 1X phosphate buffered saline (PBS) and 0.02 Sodium Azide), *S*. Typhi ISP 1820 labeled with Pacific Blue dye (*S*. Typhi-PB) were added. Bacteria were labeled as previously described (52) and 1 µl of labeled bacteria (O.D. = 0.4) was diluted in 100 µl of FC buffer and added to the cells for 30 min (RT). Cells were then washed twice with FC buffer, followed by blocking with a mixture of mouse IgG (5 μg in 25 µl of FC buffer) (Meridian Life Sciences, Memphis, TN, USA) and human IgG (5 μg in 25 µl of FC buffer) (Sigma, St Louis, MO) for 10 minutes on ice. Cells were then stained with various antibody cocktails prepared in FC buffer. After washing cells twice with FC buffer, stained cells were fixed with 1% PFA in PBS. In experiments that involve the measurement of intracellular targets, following surface staining, the cells were fixed with IC Fix buffer (Invitrogen) (30 min RT) and following washes (2x) with 1X Permeabilization Buffer (Invitrogen), cells were stained. The intracellular antibody cocktail was prepared in 1X Permeabilization Buffer. Monoclonal antibodies (mAbs) against the following molecules conjugated to the stated fluorochromes were used: CD257-FITC (clone 1D6; eBioscience), CD354-PE (clone TREM-26; BioLegend), CD16-PE-CF594 (clone 3G8; Beckton Dickinson -BD-), CD86-PerCP-Cy5.5 (clone 2331; BD), CD284-PE-Cy7 (clone Y1/82A; eBioscience), HLA-DR-BV570 (clone L243; eBioscience), CD163-BV605 (clone GHI/61; BioLegend), CD14-QDot655 (clone TuK; Invitrogen), CD274-BV711 (clone 29E.2A3; BioLegend), CD285-Alexa Fluor 647 (clone 624915; BD); CD3-Alexa Fluor 700 (clone UCHT1;BD), CD19-Alexa Fluor 700 (clone HIB19; BioLegend), CD36-APC-H7 (clone 5-271; BioLegend), and Integrin α4β7-Alexa Fluor 647 (clone ACT-1; in-house labeled). Antibodies to the next intracellular targets were used: COX-2-PE (clone L128; BD) and TNF-α-BV711 (clone MAb11; BioLegend). Samples were collected in a custom LSRII flow cytometer (BD, USA). Samples were analyzed using FlowJo (Tree Star, San Francisco, CA).

#### Gene expression

For gene expression analyses, monocytes were enriched using a negative selection kit (StemCell Technologies, Vancouver, Canada). Enriched monocytes (1x10^5^) cells were then plated in 1mL of serum-free media (DMEM, 1% L-glutamine, 1% Penicillin/Streptomycin) supplemented with 5% human AB serum. Cells were incubated for 24h (37°C in 5% CO2) and then total RNA was isolated using TriPure according to manufacturer’s protocol. Transcripts for *PTGS2* and *MRC1* were assessed by qRT-PCR (ABI Prism 7900 Sequence Detection System and software; Applied Biosystems) as previously described (Shirey MI 2010). The following primer sets were used in these studies: *HPRT* forward (5’-GTTATGGCGACCCGCAG-3’); *HPRT* reverse (5’-ACCCTTTCCAAATCCTCAGC-3’); *PTGS2* (COX-2) forward: (5’- CCCATGTCAAAACCGAGGTG -3’); *PTGS2* (COX-2) reverse (5’- CCGGTGTTGAGCAGTTTTCTC -3’); *MRC1* forward (5’- TCCTGGTTTTTGCCTCTGTC -3’); *MRC1* reverse (5’- GCACTGGGACTCACTGCAT -3’).

### Statistical methods

Differences in the frequencies (percentages) of monocyte subsets at day 0 were analyzed using parametric t-tests (2-sided, paired). Comparisons of marker expression (percentages or mean fluorescence intensity -MFI-) at day 0 between the diverse monocyte subsets were also performed using parametric t-tests (2-sided, paired). In this manuscript, as all TD volunteers had a slightly different timeframe until typhoid diagnosis, we decided to create an artificial timepoint encompassing the average of 48h-TD, 96h-TD and the time point immediately before, for each volunteer. Therefore, this artificial timepoint included the day at which the criteria for typhoid diagnosis were reached. We named this combined timepoint, Time-of-Disease (ToD) to differentiate from time of diagnosis. Changes in expression of markers in TD volunteers following challenge compared to day 0 (pre-challenge) with the average MFIs, percentages or transcript fold-changes at ToD were done using parametric t-tests (2-sided, paired). In TD volunteers, the more profound changes in marker expression were noted during ToD. Comparisons of marker expressions at steady state (day 0) between TD and NoTD volunteers were also assessed using parametric t-tests (2-sided, unpaired). Comparisons of the cell clusters rendered by UMAP, X-shift and Cluster Explorer, between Day 0 and 48h-TD, were done using unpaired, parametric, 2-sided t-tests. All statistical analyses were performed in GraphPad Prism software (GraphPad Software Inc., USA). P values <0.05, without adjustment for multiple comparisons, were considered statistically significant.

## Results

### Monocyte subset frequencies after *S*. Typhi challenge

**Figure 1:**
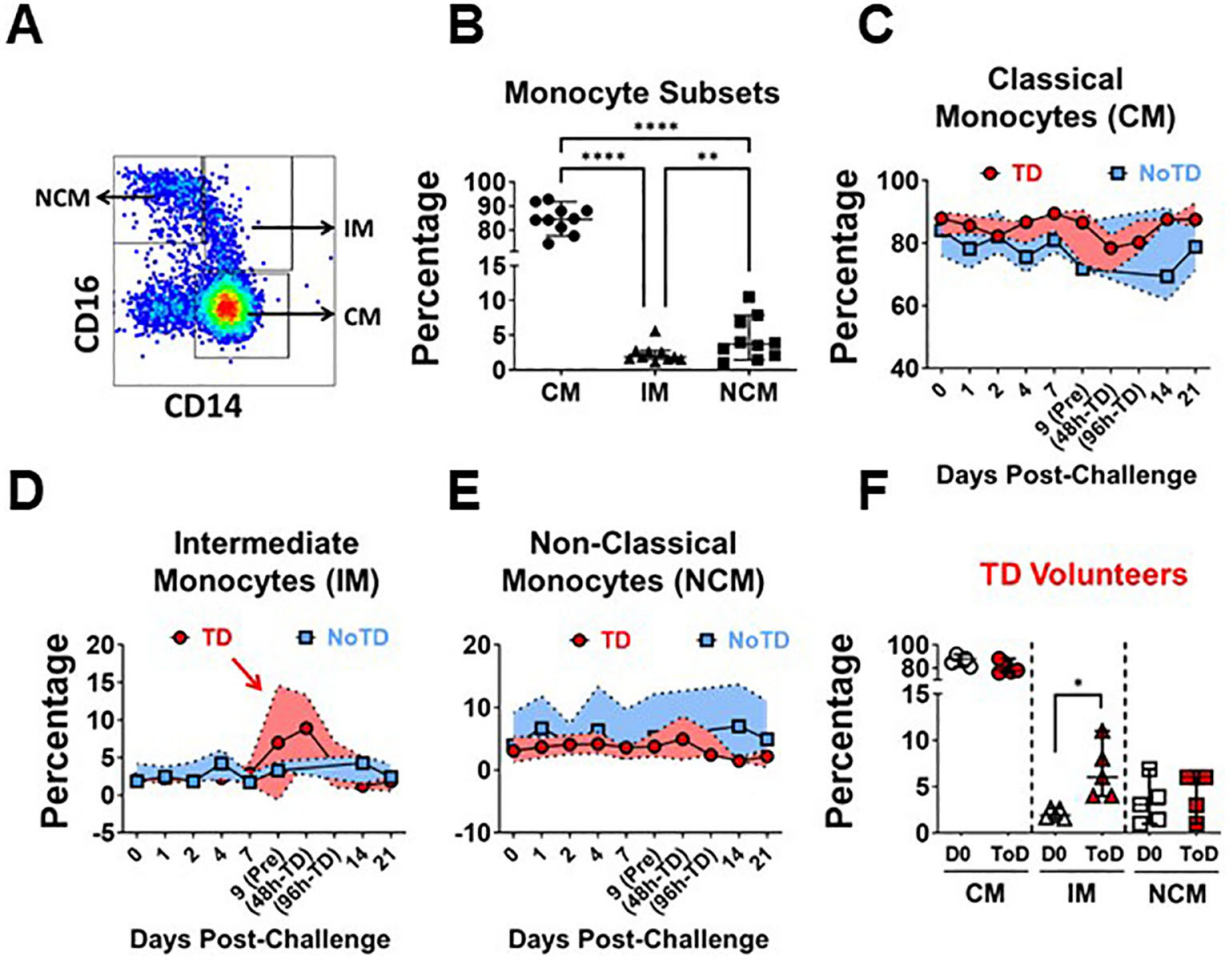
Frequency (percentage) of monocyte subsets before and after wt *S.* Typhi challenge. **(A)** displays the three circulating monocyte subsets identified by CD14 and CD16. **(B)** shows the percentage distribution of these subsets in the volunteers studied. **(C–E)** show the percentages of the populations overtime for CM, IM and NCM, respectively, in TD (red) and NoTD (blue) volunteers. **(F)** shows the comparison of the percentage of the monocytes subsets between Day 0 (pre-challenge) vs. Time-of-Disease (ToD). CM, Classical Monocytes; IM, Intermediate Monocytes; NCM, Non-classical Monocytes; ToD, Time of disease. Plots **(B, F)** show the Median and 95CI. **(C–E)** show median and interquartile range. The p values were calculated using paired t-tests (2-sided). *p<0.05, **p<0.005, ****p<0.0001.

In humans, CD14 and CD16 define three monocyte subsets in circulation, which include Classical (CM; CD14+ CD16-), Intermediate (IM; CD14+ CD6+) and Non-Classical (NCM; CD14- CD16+) Monocytes (**Figure 1A**; **Supplementary Figure 1A**). Consistent with the literature, in the volunteers participating in this study, CM comprised around 80% of the monocytes, while IM and NCM each encompassed 5-10% of the monocyte subsets (**Figure 1B**). Before wt *S.* Typhi challenge (Day 0), TD and NoTD volunteers showed similar percentages of monocyte subsets (**Supplementary Figures 1B–D**). Following challenge, TD volunteers were typhoid diagnosis criteria between days 9 and 14. Of note, every volunteer showed a slightly different time frame in which they reached diagnosis criteria. TD volunteers provided additional blood samples at 48- and 96-hours post-typhoid diagnosis (48h-TD and 96h-TD, respectively). We used these two time points plus the one immediately previous, to align the data. In NoTD volunteers, days 9 and 14 were aligned with the Pre-TD and the data point immediately after 96h-TD, respectively. These alignments were used to visualize the TD and NoTD data simultaneously, as shown in **Figures 1C–E**, which display the percentage of monocytes subsets. IM of TD volunteers showed a clear increase in their percentage between Pre-TD, 48h-TD and 96h-TD timepoints (**Figure 1D**) while the other two subsets showed limited variations (**Figures 1C, E**). NoTD volunteers showed minimal changes in the percentages of any monocyte subset. To determine whether the increase of IM in TD volunteers was statistically significant, we compared pre-challenge data (Day 0) to a data point that averaged 48h-TD, 96h-TD and the time point immediately before. This artificial timepoint encompassed the day at which the criteria for typhoid diagnosis were reached. This combined timepoint was named Time-of-Disease (ToD) to differentiate it from time of diagnosis (examples are shown in **Supplementary Figures 1E–H**). We observed significant increases in the frequencies of IM during ToD (**Figure 1F**). In contrast, no differences in the percentages of monocytes subsets were observed in NoTD volunteers (**Supplementary Figure 1I**).

### Changes in pattern recognition receptors by monocyte subsets

**Figure 2:**
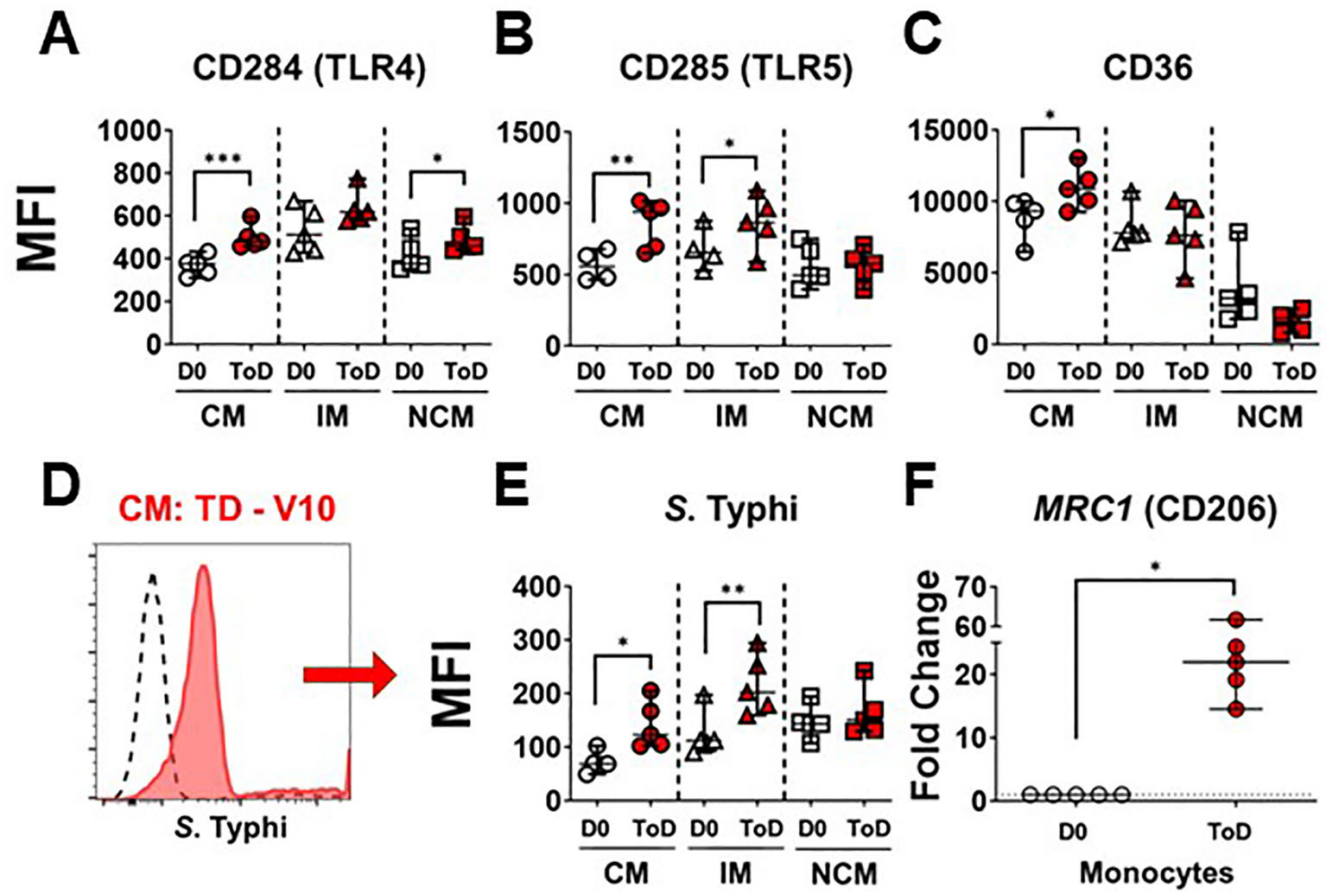
Expression of PRRs in circulating monocyte subsets after wt *S.* Typhi challenge in TD volunteers. **(A–C)** show changes in expression (MFI) of CD284 (TLR4), CD285 (TLR5) and CD36, respectively, after challenge in TD volunteers. The data compares D0 vs. ToD in the monocyte subsets. **(D)** displays an overlay of CM binding *S.* Typhi at baseline (dotted line histogram) and ToD (ref filled histogram) as an example. **(E)** displays compiled data of *S*. Typhi binding by the diverse monocyte subsets in TD volunteers. **(F)** shows the fold change of MRC1 during ToD by enriched monocytes from TD volunteers. MFI, Median Fluorescence Intensity; CM, Classical Monocytes; IM, Intermediate Monocytes; NCM, Non-classical Monocytes; ToD, Time of disease. All panels show the median and 95 CI. The p values were calculated using paired t-tests (2-sided). *p<0.05, **p<0.005, ***p<0.001.

Studies have shown differential gene transcription and protein expression profiles between the monocyte subsets (38, 39, 41, 53–55); however, little is known about changes induced by *S*. Typhi infection. Importantly, during *S*. Typhi hematic dissemination, particularly during the second bacteremia with coincides with febrile illness, the bacteria are likely to encounter blood monocytes. Therefore, we assessed receptors that play a role in the interaction with bacteria, including CD284 (TLR4), CD285 (TLR5) and CD36 (scavenger receptor). Expression of these markers was assessed in all monocyte subsets before (Day 0; **Supplementary Figures 2A–C**) and after challenge. Following wt *S.* Typhi challenge, changes in the expression of these markers among TD volunteers were predominant during ToD. CM showed a significant upregulation of CD284, CD285 and CD36. IM showed a significant upregulation of CD285 and NCM an upregulation of CD284 (**Figures 2A–C**). Upregulation of these molecules suggested that blood monocytes, particularly CM, could have an enhanced ability to bind *S*. Typhi during the disease days. To determine whether this was the case, we labeled *S*. Typhi (killed) with a fluorescent dye and exposed monocytes to these bacteria. CM and IM showed enhanced binding capacity for the *S*. Typhi during ToD, while NCM did not (**Figures 2D, E**). **Supplementary Figure 2D** shows differences in avidity for S. Typhi by the monocyte subsets at baseline. CD206 mediates non-opsonic phagocytic uptake of a wide variety of microbes including yeast, fungi, protozoa, and bacteria. This receptor has been shown to bind several bacterial molecules such as LPS and polysaccharides (56). CD206 was assessed by qRT-PCR (*MRC1 gene*) in enriched monocytes from TD volunteers and we observed enhanced *MRC1* expression during ToD (**Figure 2F**). The results suggested that circulating monocytes of TD volunteers, particularly CM, were more prone to interact with *S*. Typhi during ToD through modulation of the expression of various receptors.

### Inflammation and regulatory markers in monocyte subsets

**Figure 3:**
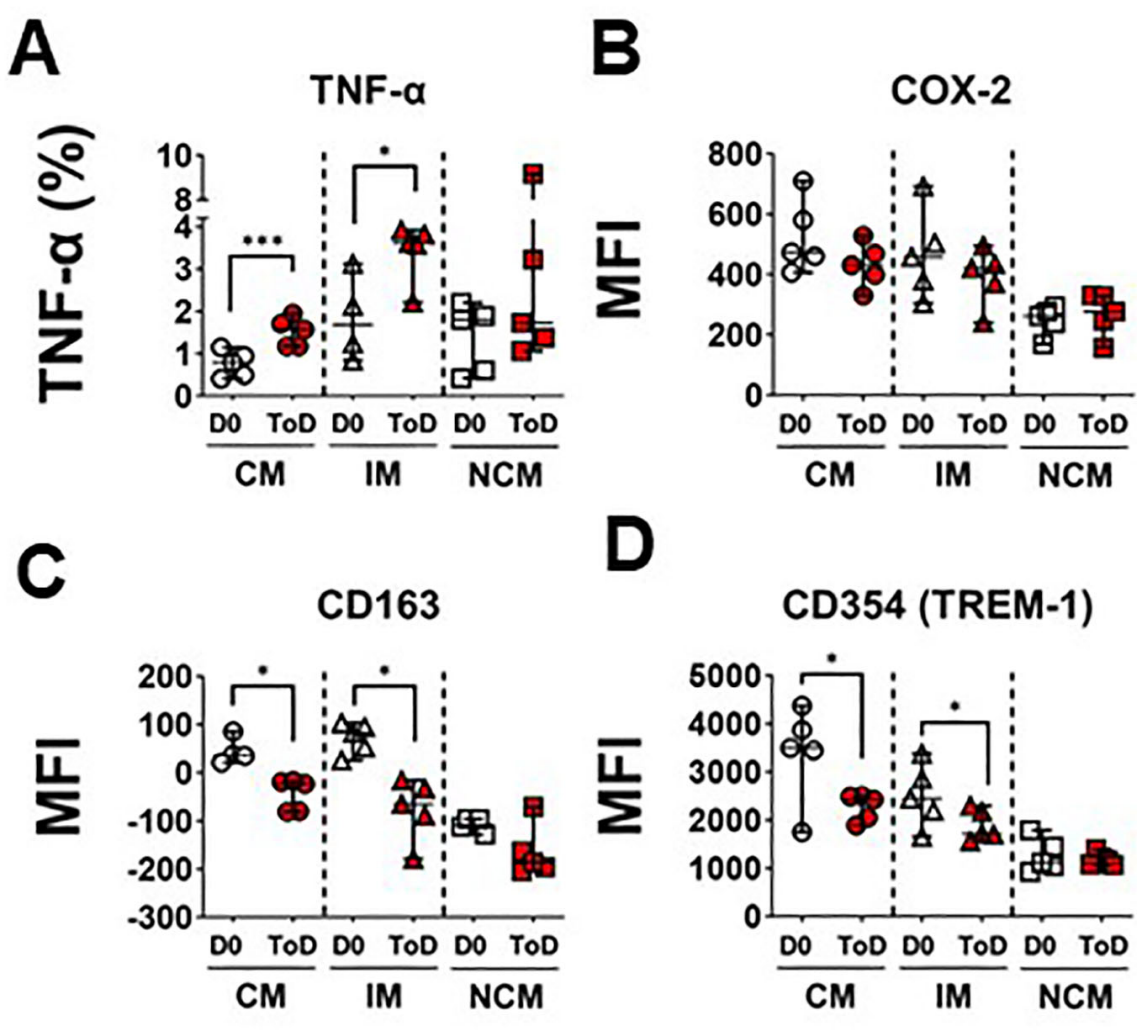
Markers of inflammation and their regulation in circulating monocyte subsets after wt *S.* Typhi challenge in TD volunteers. **(A)** displays changes in the percentage of cells producing TNF-α at baseline (D0) and ToD in circulating monocyte subsets in TD volunteers. **(B)** displays expression of COX-2 (MFI) at day 0 and during ToD. **(C)** shows changes in the expression of CD163 between D0 and ToD in TD volunteers. **(D)** shows changes in the expression of CD354 (TREM-1) between D0 and ToD in TD volunteers. MFI, Median Fluorescence Intensity; CM, Classical Monocytes; IM, Intermediate Monocytes; NCM, Non-classical Monocytes; ToD, Time of disease. All panels, except **(A)** show the median and 95 CI. The p values were calculated using paired t-tests (2-sided). *p<0.05, ***p<0.001.

The enhanced avidity for *S*. Typhi by CM and IM during ToD in TD volunteers prompted us to investigate whether these cells also exhibited upregulation of inflammatory markers by assessing expression of TNF-α production following wt challenge. The percentage of CM and IM producing this cytokine increased significantly during ToD (**Figure 3A**; **Supplementary Figure 3F**). It is worth noting that at steady state IM produced significatively more TNF-α than CM or NCM (**Supplementary Figure 3A**). We also investigated expression of COX-2, another key inflammatory marker. Contrary to the expected, after wt *S.* Typhi challenge, the expression of COX-2 did not change in any of the monocyte subsets in TD volunteers during ToD (**Figure 3B**). We confirmed these results by qRT-PCR in enriched monocytes from TD volunteers and showed that *PTGS2* (COX-2) transcripts did not increase during ToD (**Supplementary Figure 3E**). Basal levels of COX-2 expression in the diverse monocyte subsets are shown in **Supplementary Figure 3B**.

We also investigated whether mechanisms that monocytes use to limit dysregulated inflammation were activated. For this, we assessed surface expression of CD163 after challenge. In monocytes, CD163 promotes bacteria-induced pro-inflammatory cytokine production; however, extracellular TLR activation leads to shedding of CD163 as soluble CD163 (sCD163). Shedding of this molecule may be a mechanism to decrease acute and severe monocyte activation and inflammation. Interestingly, following wt *S.* Typhi challenge, surface expression of CD163 was reduced on CM and IM monocytes during ToD (**Figure 3C**). Moreover, since the mechanism of CD163 cleavage from the surface is mediated by TLR activation, we investigated whether there was downregulation of receptors involved in amplification of the TLR signal, such CD354 (TREM-1). After wt *S.* Typhi challenge, the expression of CD354 was significantly reduced in CM and IM during ToD (**Figure 3D**). Steady state expression of CD163 and CD354 is shown in **Supplementary Figures 3C, D**, respectively. Taken together, the data suggest that in TD volunteers during ToD *S*. Typhi induced expression of inflammatory markers in CM and IM, but inflammation was controlled, at least in part, by mechanisms involved in dampening the inflammatory response.

### Interaction with cells of the adaptive immune response and homing to GALT

**Figure 4:**
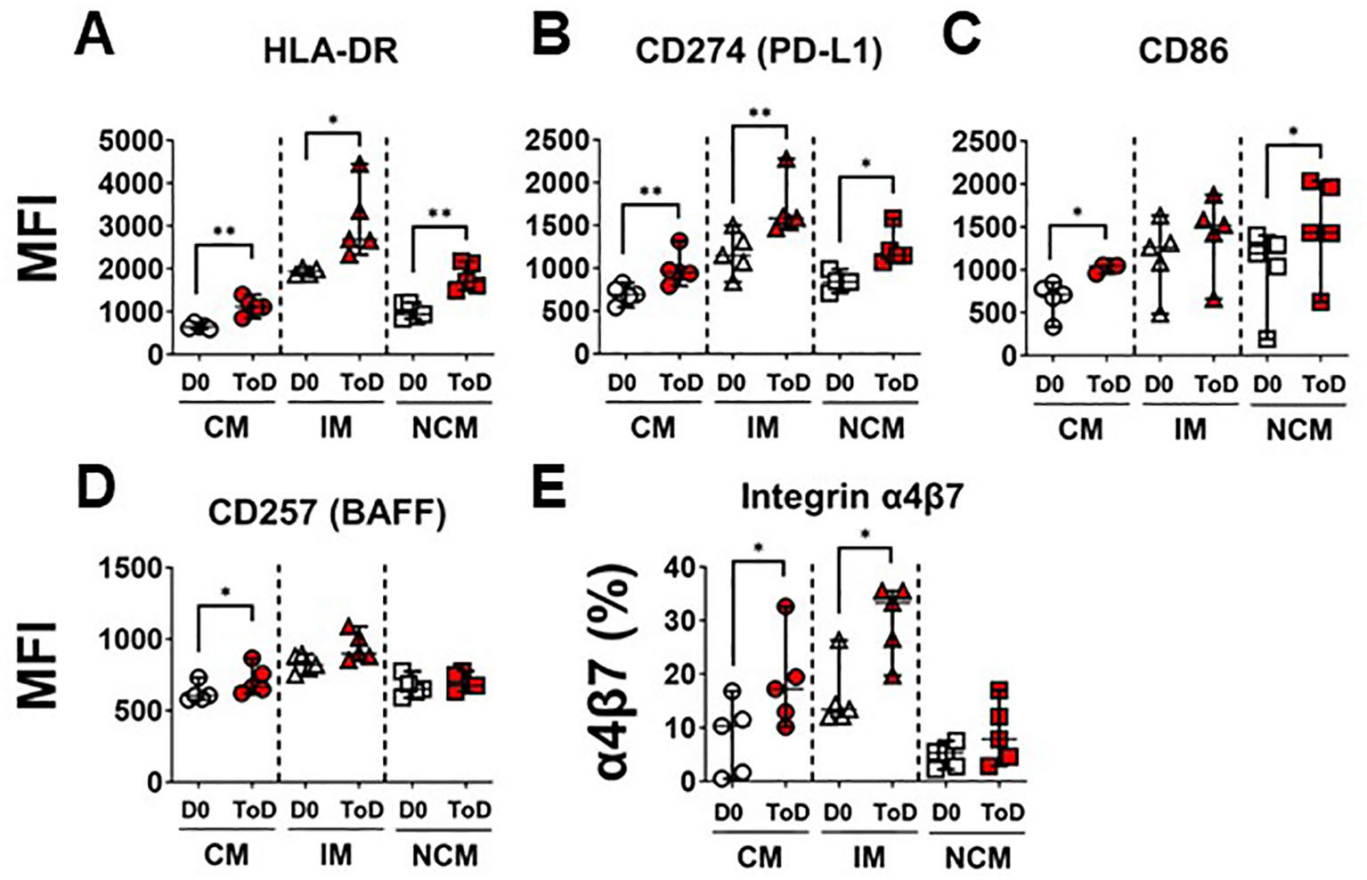
Monocyte receptors for interactions with T and B cells. **(A–C)** display changes in expression (MFI) of monocyte receptors that interact with T cells, including HLA-DR **(A)**, CD274 **(B)** and CD86 **(C)**. **(D)** shows changes in the expression (MFI) of CD257, which monocytes use to interact with B cells. **(E)** display changes in expression (percentage) integrin α4β7. Changes are shown between D0 and ToD in TD volunteers. MFI, Median Fluorescence Intensity; CM, Classical Monocytes; IM, Intermediate Monocytes; NCM, Non-classical Monocytes; ToD, Time of disease. All panels, except **(A)**, show the median and 95 CI. The p values were calculated using paired t-tests (2-sided). *p<0.05, **p<0.005.

TD volunteers showed induction of an inflammatory response by CM and IM during ToD, albeit controlled, as suggested by the reduction in the expression of CD163 and CD354. This also suggested that monocytes were being activated and likely upregulated molecules involved in their interaction with cells of the adaptive immune response. Therefore, we assessed expression of proteins involved in interaction with T cells including HLA-DR, CD274 (PD-L1) and CD86 (**Figure 4**). Upregulation of these proteins is associated with activation of antigen presenting cells, including monocyte/macrophages. Even though CD274 (PD-L1) is associated with regulation of T cell function, this protein is highly upregulated during the acute infection phase and therefore considered an activation marker (57). Following wt challenge, all the monocyte subsets in TD volunteers upregulated these molecules, except for CD86 by IM. (**Figures 4A–C**). Baseline expression levels of these proteins in the monocyte subsets are shown in **Supplementary Figure 4A–C**. At baseline (D0) IM expressed the highest levels of HLA-DR, CD274 and CD86, followed by NCM. Conversely, CM expressed the lowest levels of these receptors. We also assessed expression of CD257 (BAFF), which allows monocyte interaction with B cells. After challenge, this molecule was significantly upregulated by CM during ToD (**Figure 4D**). Baseline expression of CD257 is shown in **Supplementary Figure 4D**. At baseline, IM monocytes showed the highest expression of this marker, followed by NCM and CM. Taken together, these data suggest that monocytes, particularly CM were activated and ready to interact with T and B cells.

As indicated above, CM leave circulation and migrate to various tissues to become terminally differentiated cells. Pathogens that initially infect and gain access through the gut mucosal, like *S.* Typhi, are expected to elicit migration of activated immune cells to gut associated lymphoid tissues (GALT). Therefore, we assessed expression of integrin α4β7 by the monocyte subsets. Integrin α4β7 binds to the Mucosal vascular Addressin Cell Adhesion Molecule-1 (MAdCAM-1), which is expressed on Peyer patches, mesenteric lymph nodes, and lamina propria venules; therefore, facilitating the migration of immune cells to GALT. Following challenge, CM and IM of TD volunteers increased expression of this homing molecule suggesting that these cells are primed to migrate to the GALT (**Figure 4E**; **Supplementary Figure 4F**). Baseline expression of integrin α4β7 is shown in **Supplementary Figure 4E**. At baseline, IM had the highest frequency of cells expressing integrin α4β7.

**Figure 5:**
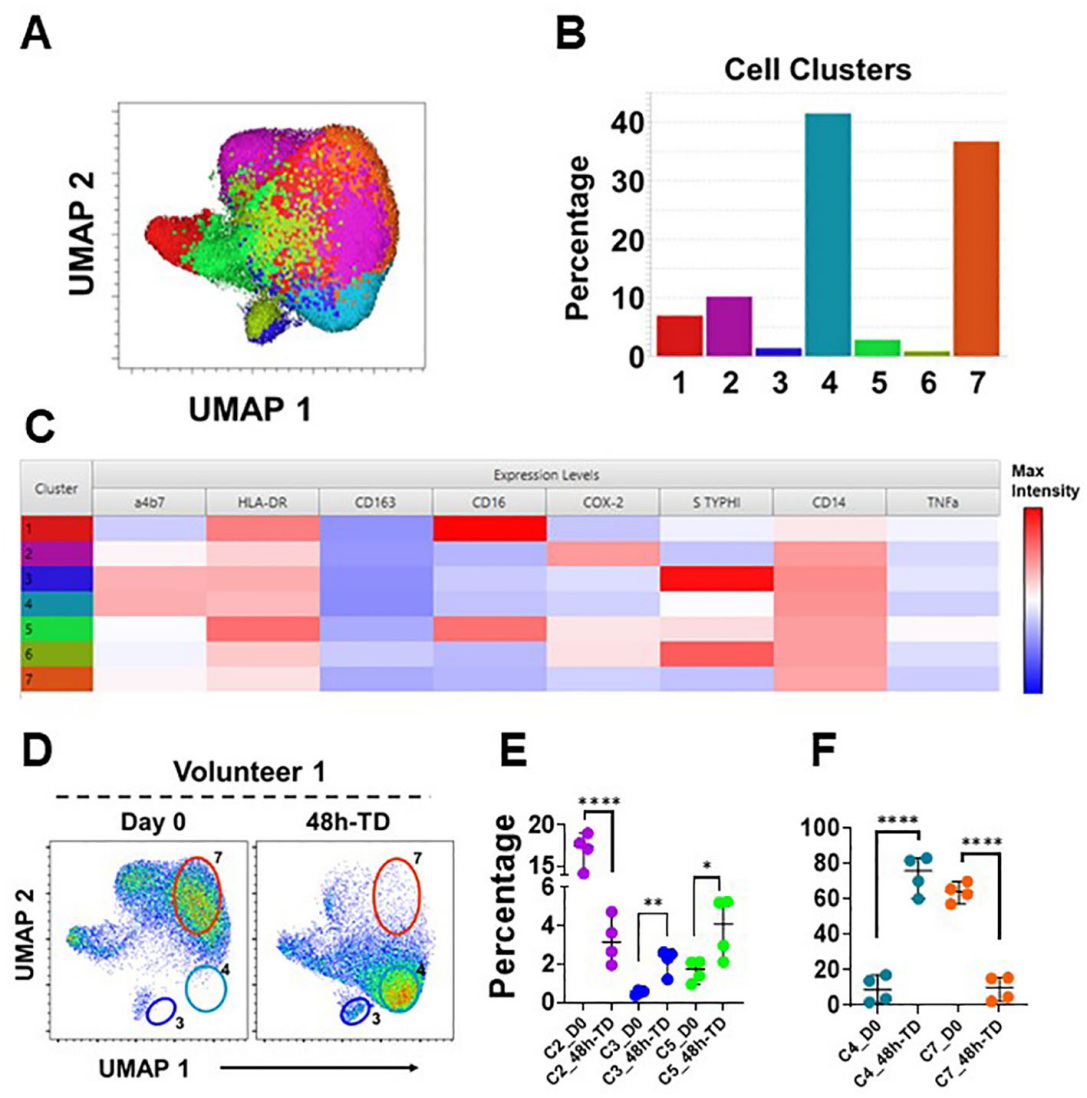
Unsupervised analysis. **(A)** shows a UMAP representation of monocytes (Day 0 and 48h-TD concatenated from 4 TD volunteers) overlayed with the seven cell clusters identified by X-Shift. **(B)** displays the size (percentage) of the clusters and **(C)** shows their composition and expression intensity of the markers in each cluster. **(D)** shows the UMAP deconvoluted on a representative volunteer at day 0 (left) and 48h-TD (right). The position of some clusters (3, 4 and 7) is shown to highlight the differences between these timepoints. **(E)** shows the changes in size (percentage) of clusters 2, 3 and 5 between Day 0 and 48h-TD. **(F)** shows similar changes, but in clusters 4 and 7. **(E, F)** show the median and 95 CI. The p values were calculated using unpaired t-tests (2-sided). *p<0.05, **p<0.005, ****p<0.0001.

To further explore the immunophenotypic characteristics of circulating monocytes, we performed unsupervised analyses in concatenated samples from Day 0 and 48h-TD from four TD volunteers. Timepoint 48-TD was selected because this showed the most significant changes among TD volunteers. This analysis included the 3 monocytes subsets (monocyte population). Samples from one volunteer where not included as one marker was omitted by mistake during staining and therefore could not be concatenated appropriately (**Figure 5**). Cells clusters rendered by a dimensionality reduction analysis (UMAP) were assessed using X-shift (**Figure 5A**). Seven cell clusters were identified (**Figure 5B**) and the marker expression composition assessed using Cluster Explorer. NCM and IM were represented in clusters 1 and 5, respectively, and all other clusters were part of the CM population (**Figure 5C**). Concatenated samples were deconvoluted to determine the clusters present on the UMAP at day 0 and 48h-TD for each volunteer. **Figure 5D** shows data from one representative volunteer highlighting prominent changes. For example, cluster 7 was present at day 0, but not at 48h-TD. On the other hand, clusters 3 and 4 were not present at day 0 but predominated at 48h-TD. The frequency (percentage) of clusters at day 0 and 48h-TD for each volunteer was compared. Clusters 3, 4 and 5 increased significantly in frequency at TD-48h, while clusters 2 and 7 decreased (**Figures 5E, F**). Clusters 3 and 4 were part of the CM population, appeared at 48h-TD, showed an activated phenotype (HLA-DR high and CD163 low) and high capacity to migrate to GALT (high integrin α4β7 expression). Of these clusters, clusters 3 and 6 (conventional monocytes) showed high binding for *S*. Typhi. Clusters 1 and 6 did not show changes between day 0 and 48-TD (**Supplementary Figure 6H**). Overall, these data show that monocyte responses to *S*. Typhi infection are very dynamic and involve CM and IM. The data suggest that CM are the main population involved in these responses and that defined CM-subsets are likely to be the dominant, but this aspect needs to be better defined in future studies. IM were involved in responses to *S.* Typhi during ToD to a lower extent than CM.

## Discussion

*S*. Typhi is a human restricted pathogen and the agent responsible for typhoid fever. These bacteria enter the host via the intestinal mucosa and after infecting local cells (gut macrophages and other phagocytes) disseminate causing a systemic disease. Circulating monocytes show great plasticity and can differentiate into various cell types (e.g., macrophages and dendritic cells -DC-) once they reach the tissues. Various monocytes subsets have been described in circulation including CM, IM and NCM. The re-establishment of a *S*. Typhi model has allowed to study in detail several arms of the immune system in those volunteers that reached typhoid diagnosis (TD) criteria. Importantly, typhoid fever reaches the blood seeding reticuloendothelial organs (primary bacteremia) and after ~8-14 days incubation, causes a secondary bacteremia that coincides with febrile illness. Bone marrow is part of the reticuloendothelial system and a site that houses monocyte precursors (mature monocytes remain in bone marrow for 2-3 days before being released into circulation). Therefore, S. Typhi can encounter monocytes either in bone marrow or in circulation. Unlike other tissues, which have a population of self-renewing tissue macrophages, all gut macrophages are derived from circulating monocytes. Therefore, we investigated whether volunteers that develop TD showed significant changes in circulating monocytes and whether there were differences within the diverse subsets. Typhoid fever causes low-level bacteremia (1-10 CFU/ml of blood) and therefore the number of monocytes that encounter the bacteria might be limited. Despite this, it is quite remarkable the profound changes noted in circulating monocytes, suggesting that the bacteria in blood are only partially responsible for these changes. It is likely that bacterial products or metabolites (derived from infected cells or other immune cells) could be involved in amplifying the monocyte activation/response.

We observed that monocyte markers involved in interaction with bacterial antigens (CD284, CD285, CD36, *MRC1*) were elevated in TD volunteers during ToD, particularly in the CM and IM subsets (**Figures 2A–C, F**). Moreover, this was associated with an enhanced ability of monocytes to bind *S.* Typhi (**Figure 2E**). The enhanced ability of monocytes to bind *S*. Typhi during ToD agrees with a previous report from our group (50). In the current study, we extended the assessments to include monocyte subsets and showed that CM and IM are the main subpopulations binding *S*. Typhi. We expected an increased expression of CD284 and CD285, as these receptors recognize LPS and flagellin, both present in *S.* Typhi. Less clear was the role of CD36, a scavenger receptor. CD36 facilitates innate immune responses against gram-positive bacteria such as *S.* aureus and *S. pneumoniae*. However, the role of CD36 in the defense against Gram negative bacteria has been less evident (58–61). It was recently shown that the absence of this receptor significantly increased mice susceptibility to acute intrapulmonary infection by *K. pneumonia*, suggesting a role for CD36 in controlling infections to Gram-negative bacteria (62). Our data provides evidence that CD36 also plays a role during *S.* Typhi infection, another Gram-negative pathogen (**Figure 2C**).

Another important molecule for monocytes/macrophages is CD206. CD206 recognizes and binds a wide range of ligands, including peptide hormones, lysosomal hydrolases, mannose, fucose, and collagen, along with allergens and microbial products including CpG DNA. CD206 also mediates the non-opsonic phagocytic uptake by macrophages of a wide variety of microbes including yeast, fungi, protozoa, and bacteria. In the case of bacterial infections, it is known that CD206 binds LPS and polysaccharides (56). Moreover, soluble CD206 (sCD206) has been identified in the ascites fluid from cirrhosis patients with spontaneous bacterial peritonitis and is considered a marker of macrophage activation in these cases (63). To our knowledge, no data regarding the role of this molecule in *S.* Typhi infection has been reported. Herein we show that enriched monocytes from TD volunteers increased expression of *MRC1* (CD206), probably the result of interactions with antigens from the bacteria, further suggesting activation of these cells.

TD volunteers showed evidence that *S.* Typhi induced inflammation, as the percentage of CM and IM producing TNF-α was upregulated during ToD. However, it appears that factors involved in controlling inflammation were also at play since CD163 was downregulated during ToD. CD163 is a member of the scavenger receptor cysteine-rich (SRCR) superfamily class B, is exclusively expressed by monocytes and macrophages and is able to bind both Gram-positive and Gram-negative organisms (64). Extracellular TLR activation leads to shedding of CD163 as soluble CD163 (sCD163). Because surface CD163 on macrophages functions as an innate immune receptor for bacteria (64), its shedding may be a mechanism to decrease acute and severe monocyte activation and inflammation. Several observations support this contention. For example, physiological doses of LPS acutely stimulate CD163 shedding within 1 hour, followed by increased expression on the surface of monocytes and macrophages 24 hours later (65–67). Moreover, levels of CD163 on monocytes are inversely correlated with the levels of sCD163 found *in-vitro* in tissue culture medium and *in-vivo* in plasma (65). Therefore, the decrease in CD163 expression by CM and IM (**Figure 3C**) in TD volunteers during ToD most likely reflects activation of mechanisms to control the inflammation, possibly triggered by the engagement of TLR4/5 by *S.* Typhi. Furthermore, since the mechanism of CD163 cleavage from the surface is mediated by TLR activation, we assessed whether receptors involved in amplification of TLR signals (e.g., CD354), were downregulated. We showed that CM and IM exhibited downregulation of this marker, providing further evidence that mechanisms involved in controlling inflammation were activated. The lack of increase in COX-2 expression (**Figure 3B**; **Supplementary Figure 3E**) might also be a consequence of these regulatory mechanisms.

The increased expression of HLA-DR, CD274 and CD86 by TD volunteers during ToD confirmed that these cells, particularly CM and IM, were activated and possibly ready to interact with T cells. Additionally, CM and IM showed increased expression of integrin α4β7 suggesting that these cells were migrating to gut lymphoid tissues. Integrin α4β7 upregulation is also in agreement with our previous studies (50). Additionally, these findings suggest that interaction with the bacteria or bacterial products triggered monocyte activation during the systemic dissemination of *S*. Typhi and before reaching the tissues. Interestingly, little is known about the role of CD257 in *Salmonella* infection. CD257 is a homotrimer that is found either on the cell surface as a type II transmembrane protein or is released in a soluble form after cleavage (68, 69). CD257 is required for B cell survival, maturation, activation and homeostasis and is found on neutrophils, monocytes, macrophages and dendritic cells (68, 70, 71). Monocytes are one of the major cell types expressing and secreting CD257 during infection and inflammation. A recent report showed that CD257 is important for protection from *Salmonella* infection in a mouse model (72). We showed that CM exhibited increased CD257 expression on their surface during ToD, suggesting a role for this molecule in *Salmonella* infections in humans. Future research should address this issue in more detail, including assessment of this cytokine in sera, as this molecule is ultimately released from the cells and is only transiently expressed on the surface of monocytes and other innate cells.

Unsupervised analysis showed that the CM were the main subset responding during TD, as 5 of the 7 cell clusters identified belonged to this subset. Significant changes were identified at the 48h-TD timepoint. Of note, some clusters were present only at day 0, but were either not present or their presence was greatly reduced at the 48hTD time point (e.g., Cluster 7). Other cell clusters behaved in the opposite way, such as cluster 4, which was present only at 48h-TD (**Figure 5**). Of importance is the identification of small clusters within CM, such as cell cluster 3, whose cells showed an activated phenotype, increased capacity to migrate to GALT and enhanced binding of *S.* Typhi. Future studies will be directed to investigate the role that these cells play in typhoid disease.

The data collected in this study, despite that involved only 5 TD and 5 noTD volunteers, provided consistent, significant results. Additionally, the data from TD volunteers reproduced aspects we had previously reported in the overall monocyte population (e.g., upregulation of integrin α4β7); therefore, despite the limited number of volunteers the results are consistent across studies (50). The data in the current study suggest that CM are the main monocyte population being activated by *S.* Typhi during ToD. Importantly, CM are likely the most important population since they are not only the largest monocyte subset, but also most of them appear to have the capacity to migrate to the gut tissues to differentiate into macrophages or DCs. Importantly, IM showed upregulation of most of the markers assessed, including integrin α4β7, which might reflect their transition status towards NCM. Alternatively, it might suggest that during acute infection, IM might be malleable and able to revert to CM or perform similar functions to CM. Of note, in TD volunteers, no marker showed changes compared to day 0, before day 9. It is also important to note that at baseline, each monocyte sub-population expressed diverse degrees of the molecules assessed (**Supplementary Figures 2**-**4**), which is in line with previous reports from other groups and reflect their differentiation status (38, 39, 53, 54, 73). Also, at baseline TD and NoTD volunteers showed similar expression levels of the molecules assessed (**Supplementary Figure 5** **).** Importantly, the findings in TD volunteers agree with previously published data from our group (50). In this manuscript, we have expanded the observations to include diverse monocyte subsets. One limitation of our study is its descriptive nature. Despite the fact that the human challenge model allows a detailed characterization of the immune response, it does not allow experimental immunological approaches such as depletion of cell populations before infection, cells/genes knock out/in, tissue harvesting, etc. that are common in animal models. To address this issue, we and other groups are developing systems that mimic human tissues to better characterize immunity to *S*. Typhi and other pathogens at the local level and in lymphoid tissues (74–79).

Finally, NoTD volunteers did not show any changes in the expression of the molecules assessed at any of the timepoints assessed (**Supplementary Tables 1**-**3**). **Supplementary Figure 1I** shows one example of the lack of changes in the markers assessed in NoTD volunteers. It is also important to note that in our previous study we reported that monocytes of NoTD volunteers showed enhanced binding of *S*. Typhi 24h after infection (Day 1). In the current study we did not detect this phenomenon. The size of the inoculum could be partially responsible for these differences, as in the previous manuscript we reported findings in volunteers that received 10^4^ CFU; while in the current one from subjects that received 10^3^ CFU. A lower inoculum could result in less activation of the mechanisms that lead to enhanced avidity for the bacteria in NoTD volunteers. Another possibility could involve differences in timing of the enhanced *S*. Typhi binding. If the *S*. Typhi binding peak in NoTD volunteers that received 10^3^ CFU took place earlier or later than 24h (e.g., 12h or 36h) the current blood sampling could not have captured this phenomenon. Future studies will investigate this aspect in more detail. In sum, we provide new insights into the changes that diverse circulating monocyte subsets undergo after typhoid diagnosis (TD volunteers during ToD), which might be important to control this disease since circulating monocytes, particularly CM, will ultimately become intestinal or tissue macrophages once they reach the gut or other target tissues.

## Data Availability

The original contributions presented in the study are included in the article/**Supplementary Material**. Further inquiries can be directed to the corresponding authors.
