# Supplementary material for: Changes in monocyte subsets in volunteers who received an oral wild-type Salmonella Typhi challenge and reached typhoid diagnosis criteria
Source: Front Immunol. 2024 Aug 27;15:1454857. doi: 10.3389/fimmu.2024.1454857 (PMC11388309; doi:10.3389/fimmu.2024.1454857)
Supplement: Supplementary file 1 [file DataSheet1.docx]

**Changes in monocyte subsets in volunteers who received an oral wild-type *Salmonella* Typhi challenge and developed typhoid disease**


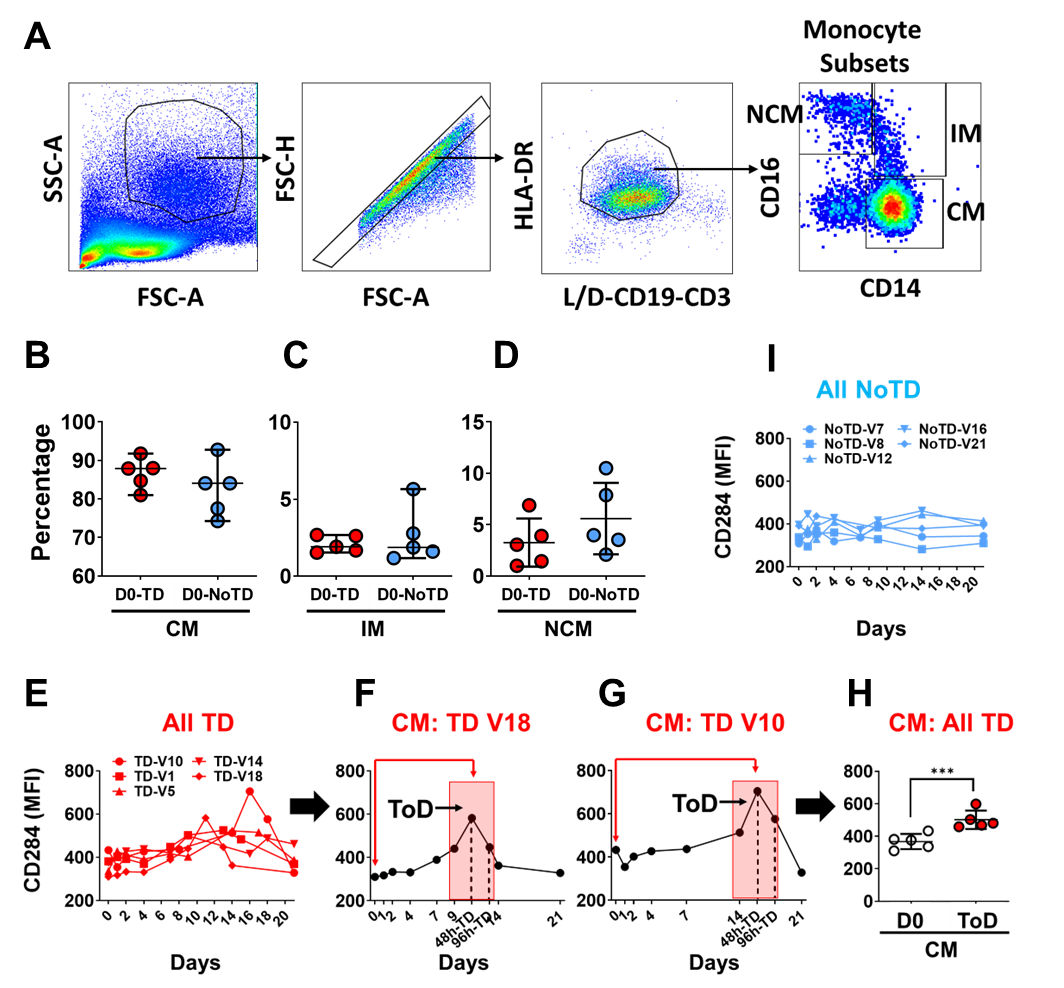
**Supplementary Information**

**Supplementary Figure 1.** Panel **A** shows the basic gating strategy used to identify monocytes and their subsets. Panels **B** to **D** show the percentages of the three circulating monocytes subsets in TD (red symbols) and NoTD (blue symbols) volunteers at day 0 (pre-challenge). No statistical differences among TD and NoTD were identified. Panel **E** shows time course changes in CD284 (TLR4) expression (MFI) in CM (all TD volunteers assessed). Panels **F** and **G** show differences in the time frame of disease development in 2 volunteers (Vols 18 and 10). The red box indicates the time-of-disease (ToD) and the red arrows indicate the comparisons performed in panel **H** (D0 vs. ToD). Panel **I** shows data from CD284 (TLR4) expression (MFI) in CM of the volunteers that did not develop disease (NoTD). The expression of CD284 did not change after challenge.


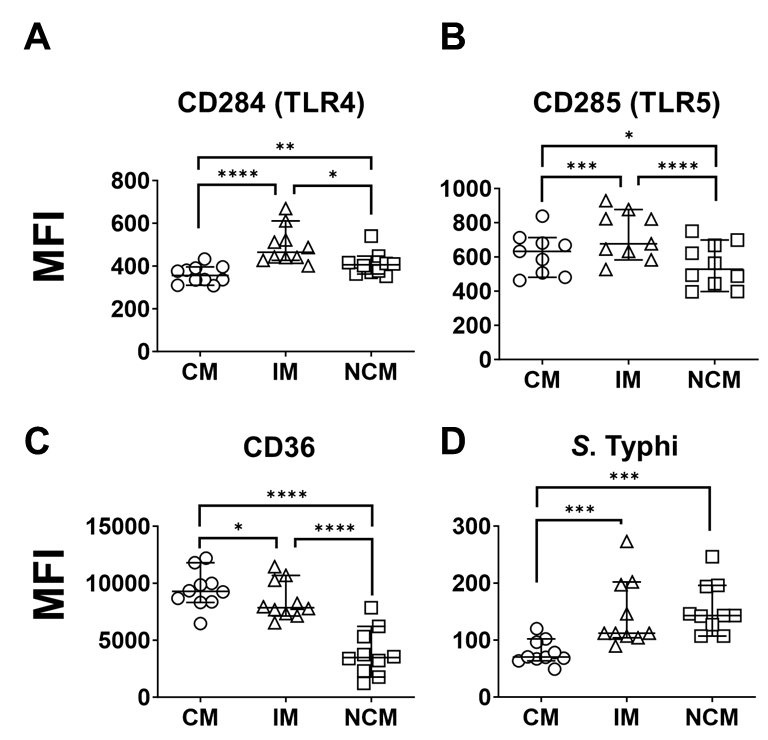


**Supplementary Figure 2. Expression of PRRs in circulating monocyte subsets at baseline.** Panels **A** to **C** show the baseline (D0) expression (MFI) of CD284 (TLR4), CD285 (TLR5) and CD36, respectively, in circulating monocyte subsets. The data displayed include all volunteers assessed. **D** displays the ability of the monocyte subsets to bind *S*. Typhi at baseline. Before challenge, IM and NCM showed a higher ability to bind *S*. Typhi than CM. MFI: Median Fluorescence Intensity. CM: Classical Monocytes, IM: Intermediate Monocytes, NCM: Non-classical Monocytes. ToD: Time of disease. All plots show the median and 95 CI. The p values were calculated using paired t-tests (2-sided). *p<0.05, ** p<0.005, *** p<0.001, **** p<0.0001.


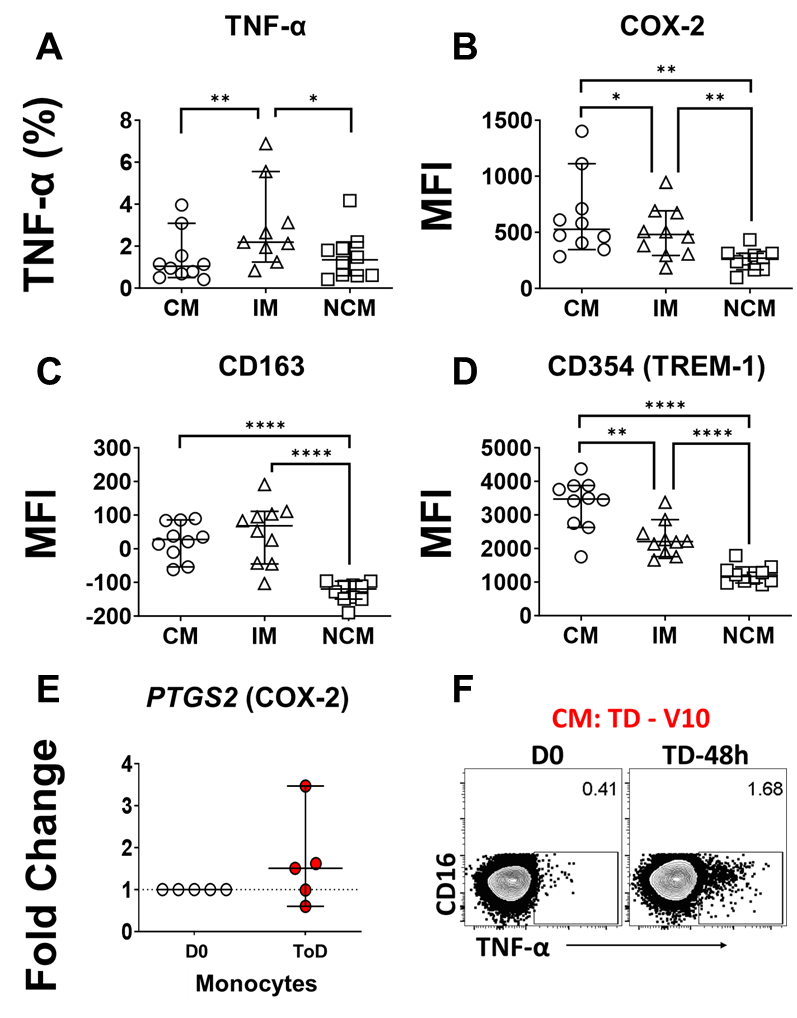


**Supplementary Figure 3. Markers of inflammation and its regulation at baseline.** Panel **A** shows the percentage of monocytes producing TNF-α at D0 in all volunteers assessed. At this timepoint, IM produced significatively more TNF-α than CM or NCM. Panel **B** displays the expression (MFI) of COX-2 at D0 showing that CM expressed the highest levels of COX-2, followed by IM and NCM. In panel **C**, the expression (MFI) of CD163 is shown. At steady state, CM and IM monocytes expressed similar levels of CD163, while NCM expressed significantly lower levels of this molecule. In **D** we show the expression (MFI) of CD354 at day 0. CM expressed the highest levels of this molecule, followed by IM and NCM. Panel **E** shows *PTGS2* transcripts in enriched monocytes at D0 and ToD in TD volunteers. Panel **F** shows one example of the gating strategy used to determine TNF-α production by CM. The data shown is from one representative TD volunteer and underscores the changes in expression of this marker between D0 and TD-48h. MFI: Median Fluorescence Intensity. CM: Classical Monocytes, IM: Intermediate Monocytes, NCM: Non-classical Monocytes. ToD: Time of disease. All plots, except **A**, show the Median and 95 CI. The p values were calculated using paired t-tests (2-sided). *p<0.05, ** p<0.005, *** p<0.001, **** p<0.0001.


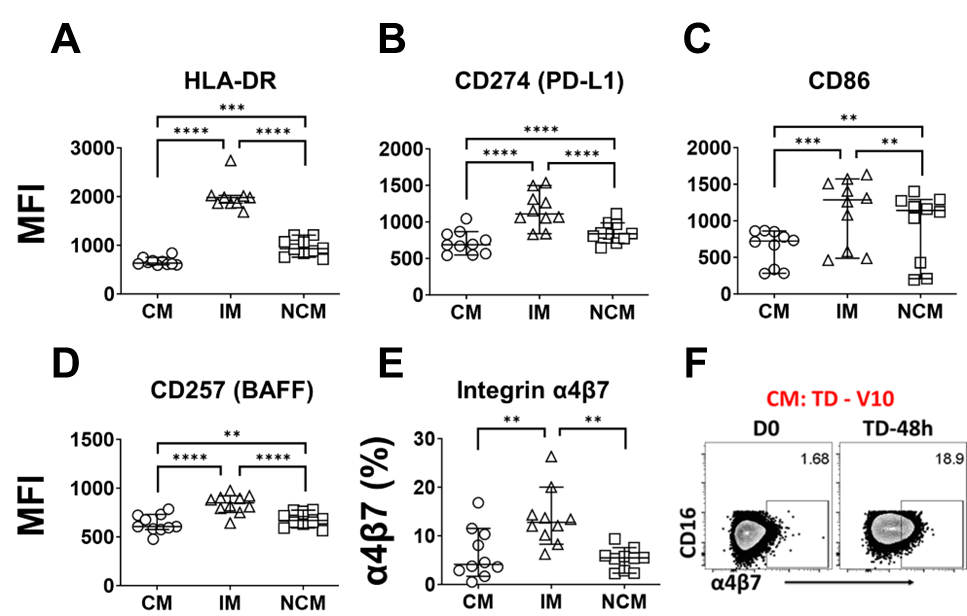


**Supplementary Figure 4. Monocyte receptors for interactions with T and B cells at baseline.** Panels **A, B** and **C** display the expression (MFI) of CD163, CD274 and CD86, respectively, at baseline in all the volunteers assessed. These receptors allow interaction of monocytes with T cells. At baseline (D0) IM expressed the highest levels of HLA-DR, CD274 and CD86, followed by NCM. Conversely, CM expressed the lowest levels of these receptors. Panel **D** shows baseline expression (MFI) of CD257. This receptor is used to interact with B cells. At baseline, IM monocytes showed the highest expression of this marker, followed by NCM and CM. Panel **E** shows the percentage of monocytes expressing integrin α4β7. At baseline, IM had the highest frequency of cells expressing integrin α4β7. Panel **F** shows an example of the gating strategy used to determine expression of integrin α4β7 in CM. The data shown is from one representative TD volunteer and highlights the changes in expression of this marker between D0 and TD-48h. MFI: Median Fluorescence Intensity. CM: Classical Monocytes, IM: Intermediate Monocytes, NCM: Non-classical Monocytes. ToD: Time of disease. All plots show the median and 95 CI. The p values were calculated using paired t-tests (2-sided). *p<0.05, ** p<0.005, *** p<0.001, **** p<0.0001.


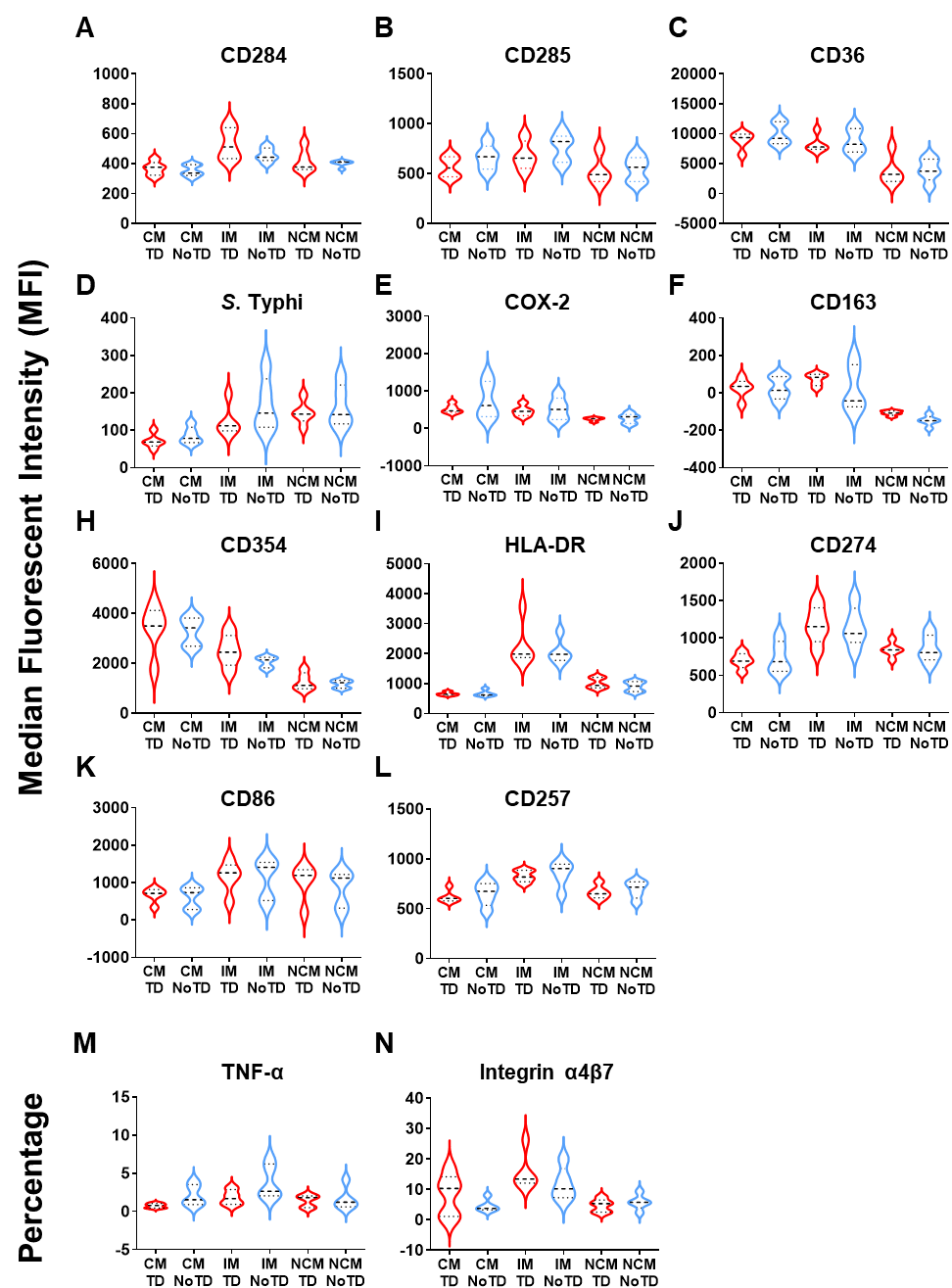


**Supplementary Figure 5. Baseline (Day 0) expression of markers in circulating monocyte subsets in TD and NoTD volunteers.** Panels **A-L** show Median fluorescent Intensity (MFI) expression of the indicated marker in monocyte subsets of participants before *S.* Typhi challenge (Day 0). Panels **M** and **N** show similar data but as percentage of the marker. Red Violin plots indicate data from TD volunteers (n=5). Blue Violin plots indicate NoTD volunteers (N=5). CM: Classical Monocytes; IM: Intermediate Monocytes, NCM: Non-classical Monocytes. No significant differences between TD and NoTD volunteers (paired t-tests) were identified at baseline for any of the markers assessed.


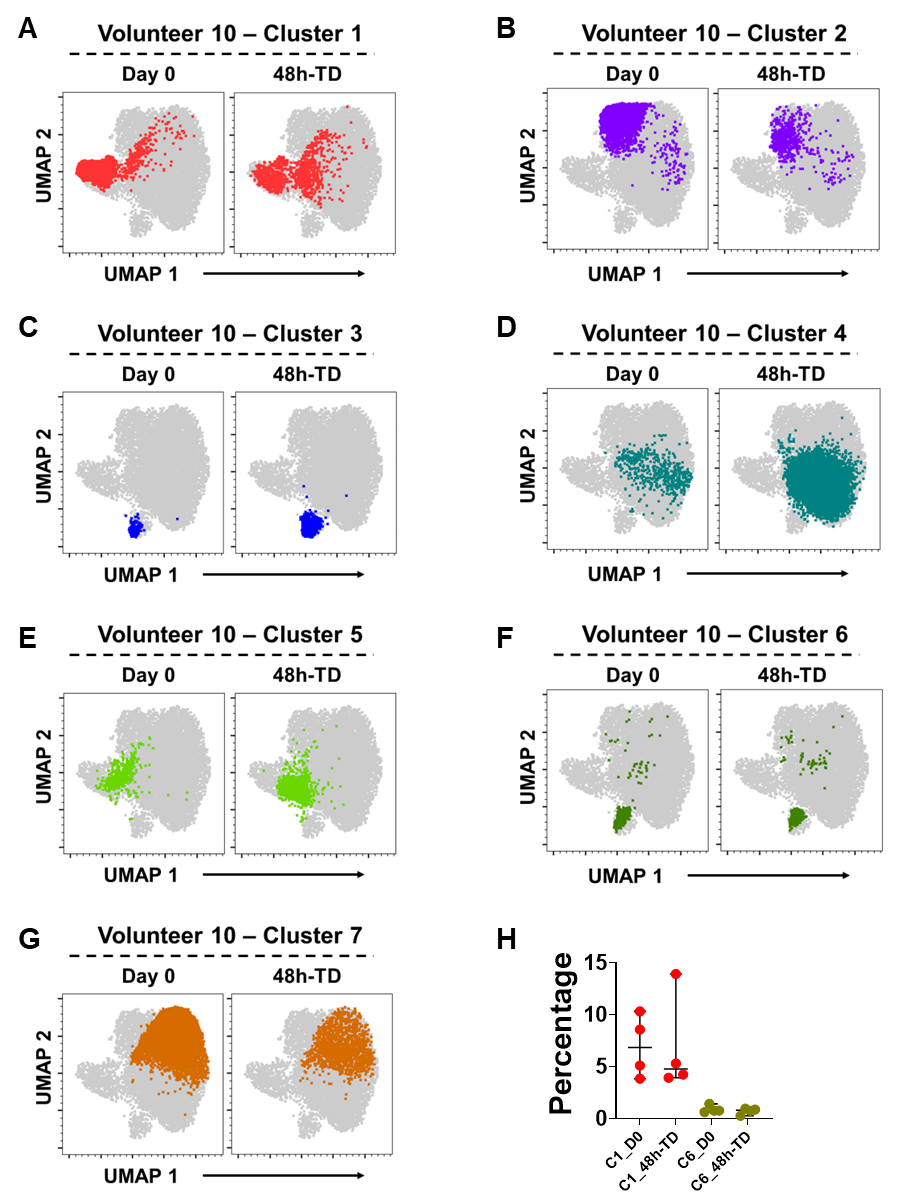


**Supplementary Figure 6. Overlay of Cell Cluster over UMAP.** Example of changes in the frequencies of cell clusters between day 0 and 48h-TD in a representative TD volunteer. **A-G** show clusters 1-7 overlaid, respectively, on the UMAP created by concatenating samples from day 0 and 48h-TD. Panel **H** displays the frequencies (percentages) of clusters 1 and 6, which did not change between day 0 and 48h-TD.

**Supplementary Table 1. Expression of Immune Markers in Classical Monocytes in NoTD Volunteers.**  The table displays the means and 95% CI of the assessed markers at days 0, 1, 2, 4, 7, 9, 14 and 21 of the study. No differences significant differences in the expression of the markers between day 0 and other study days was identified.

|  |  |  | **α4β7** | **TNF-α** | **CD163** | **HLA-DR** | **CD285** | **CD36** | **CD163** | **CD257** | ***S*. Typhi** | **CD354** | **CD284** | **CD86** | **CD274** |
| --- | --- | --- | --- | --- | --- | --- | --- | --- | --- | --- | --- | --- | --- | --- | --- |
| **Days** | **n** | **Stats Displayed** | **%** | **%** | **MFI** | **MFI** | **MFI** | **MFI** | **MFI** | **MFI** | **MFI** | **MFI** | **MFI** | **MFI** | **MFI** |
| 0 | 5 | Mean (95% CI) | 4.6 (2.8, 6.4) | 1.8 (0.9, 2.7) | 24.7 (-29.5, 78.8) | 1106 (749.7, 1462.3) | 662.6 (551.8, 773.4) | 9988.4 (8337.6, 11639.2) | 288.2 (242.2, 334.2) | 650.6 (546.1, 755.1) | 85.8 (65.9, 105.7) | 3287.6 (2785.4, 3789.8) | 353.4 (319.7, 387.1) | 603.8 (340.9, 866.7) | 741.6 (555.8, 927.4) |
| 1 | 5 | Mean (95% CI) | 6 (3.5, 8.4) | 1.3 (0.8, 1.9) | 12.5 (-59.4, 84.4) | 1099.8 (795.6, 1404) | 681.8 (572, 791.6) | 10054.6 (7910, 12199.2) | 290.8 (248.7, 332.9) | 677.8 (577.1, 778.5) | 87.6 (69.1, 106.1) | 3399.6 (2785.9, 4013.3) | 366 (318.5, 413.5) | 609.6 (318.5, 900.7) | 757.2 (577.3, 937.1) |
| 2 | 4 | Mean (95% CI) | 6.8 (4, 9.6) | 1 (0.3, 1.7) | -6.7 (-61.1, 47.8) | 918.8 (728.3, 1109.2) | 641 (562.9, 719.1) | 9379.8 (7779.2, 10980.3) | 263.5 (188.3, 338.7) | 635.8 (508.7, 762.8) | 87.5 (67.4, 107.7) | 3362.8 (2798.4, 3927.1) | 361.8 (336.4, 387.1) | 590.3 (264.8, 915.7) | 675.8 (545, 806.5) |
| 4 | 5 | Mean (95% CI) | 4.4 (2.3, 6.5) | 1.6 (0.7, 2.4) | 16 (-55.8, 87.9) | 1091 (783.4, 1398.6) | 712.8 (587.7, 837.9) | 10348.6 (8875.4, 11821.8) | 316 (259.8, 372.2) | 687.4 (560.9, 813.9) | 92.1 (75.6, 108.6) | 3761.8 (2974.9, 4548.7) | 389.8 (346, 433.6) | 668 (373.9, 962.1) | 797.6 (615.7, 979.5) |
| 7 | 3 | Mean (95% CI) | 5.5 (3, 8) | 0.9 (0.4, 1.4) | -31.9 (-53.4, -10.4) | 889.7 (634.2, 1145.1) | 640.3 (508.4, 772.3) | 9262.3 (7266.4, 11258.2) | 224 (164, 284) | 630.7 (496.2, 765.1) | 91.4 (70.7, 112.1) | 3290.7 (2975.2, 3606.1) | 350.3 (329.6, 371.1) | 639 (278, 1000) | 629.7 (533.5, 725.8) |
| 9 | 5 | Mean (95% CI) | 7.6 (3.9, 11.2) | 1.1 (0.6, 1.6) | 14.9 (-53.5, 83.3) | 1178 (846.1, 1509.9) | 723.2 (649.4, 797) | 10431.4 (8606.2, 12256.6) | 239.6 (199.3, 279.9) | 695.2 (584.2, 806.2) | 94.4 (74, 114.8) | 3133.8 (2803.8, 3463.8) | 380.8 (352.2, 409.4) | 707.6 (384.2, 1031) | 787.4 (624.8, 950) |
| 14 | 5 | Mean (95% CI) | 6.5 (3.8, 9.2) | 1.3 (0.2, 2.4) | -11.8 (-76.9, 53.2) | 1126.2 (787.7, 1464.7) | 728.2 (607.8, 848.6) | 10137.8 (6856.5, 13419.1) | 204.6 (148.3, 260.9) | 632.8 (499.4, 766.2) | 99.2 (74.7, 123.6) | 3058.2 (2508.2, 3608.2) | 380.6 (315.4, 445.8) | 695.8 (403.6, 988) | 782.4 (504.6, 1060.2) |
| 21 | 5 | Mean (95% CI) | 5.2 (1.5, 8.9) | 1.4 (0.4, 2.4) | 7 (-46.2, 60.2) | 1071.2 (729, 1413.4) | 712.4 (571.6, 853.2) | 10625.4 (7731.1, 13519.7) | 229.8 (176.7, 282.9) | 672.2 (567.7, 776.7) | 90.2 (69.5, 110.9) | 3298 (2602.7, 3993.3) | 369.8 (332.3, 407.3) | 741.6 (386.7, 1096.5) | 795.2 (552.2, 1038.2) |

|  |  |  | **α4β7** | **TNF-α** | **CD163** | **HLA-DR** | **CD285** | **CD36** | **CD163** | **CD257** | ***S.* Typhi** | **CD354** | **CD284** | **CD86** | **CD274** |
| --- | --- | --- | --- | --- | --- | --- | --- | --- | --- | --- | --- | --- | --- | --- | --- |
| **Days** | **n** | **Stats Displayed** | **%** | **%** | **MFI** | **MFI** | **MFI** | **MFI** | **MFI** | **MFI** | **MFI** | **MFI** | **MFI** | **MFI** | **MFI** |
| 0 | 5 | Mean (95% CI) | 11.7 (6.9, 16.4) | 5 (1.1, 8.9) | 22.2 (-85.9, 130.3) | 2573.6 (1502.2, 3645) | 760.4 (636, 884.8) | 8770 (6975.2, 10564.8) | 398.2 (335.3, 461.1) | 850.2 (735.4, 965) | 167.4 (105.6, 229.2) | 2052 (1857.8, 2246.2) | 458.8 (417.9, 499.7) | 1106.4 (634.1, 1578.7) | 1148.8 (916.8, 1380.8) |
| 1 | 5 | Mean (95% CI) | 10.5 (7.6, 13.4) | 3.6 (2.6, 4.6) | 4.4 (-102.3, 111) | 2718.4 (1863.9, 3572.9) | 809.8 (632.7, 986.9) | 9338.8 (6251.4, 12426.2) | 395.2 (362.3, 428.1) | 894 (806, 982) | 164.4 (104, 224.8) | 2315.2 (1913.1, 2717.3) | 473.8 (403, 544.6) | 1040.6 (582.9, 1498.3) | 1134.4 (925.8, 1343) |
| 2 | 4 | Mean (95% CI) | 7.9 (5.5, 10.3) | 2.2 (1.3, 3.2) | -13.4 (-92.4, 65.6) | 2430 (1711.8, 3148.2) | 761 (651.9, 870.1) | 7826.5 (5879.4, 9773.6) | 416.3 (336.3, 496.2) | 857.3 (711.6, 1002.9) | 162 (122.9, 201.1) | 2108.8 (1723.2, 2494.3) | 490.3 (474.8, 505.7) | 1064.5 (521.7, 1607.3) | 1096.5 (967.6, 1225.4) |
| 4 | 5 | Mean (95% CI) | 9 (7.3, 10.6) | 3.1 (1.8, 4.4) | -2.5 (-125.7, 120.7) | 2523.6 (1570, 3477.2) | 824.2 (663.2, 985.2) | 9883 (5951.4, 13814.6) | 398.6 (334, 463.2) | 853.8 (732.7, 974.9) | 185 (129.1, 240.9) | 2684.4 (1585.7, 3783.1) | 498.4 (450.8, 546) | 1044.2 (601.6, 1486.8) | 1149.4 (909.2, 1389.6) |
| 7 | 3 | Mean (95% CI) | 6.4 (5.8, 7.1) | 3.5 (0.8, 6.2) | -77.7 (-126.6, -28.8) | 2274.3 (1451.4, 3097.3) | 823.3 (571.7, 1075) | 7840.7 (5318.7, 10362.6) | 389.7 (347.3, 432) | 895 (747.3, 1042.7) | 157.3 (124.3, 190.4) | 2186.7 (1958.3, 2415.1) | 505.7 (458.2, 553.1) | 1163.3 (600.4, 1726.3) | 1106.3 (902.2, 1310.4) |
| 9 | 5 | Mean (95% CI) | 8.6 (5.6, 11.7) | 2.1 (1.7, 2.5) | -16.8 (-112.1, 78.5) | 2692.2 (1947.9, 3436.5) | 847 (686.2, 1007.8) | 8701.8 (6590, 10813.6) | 363.2 (327.6, 398.8) | 933.2 (820.2, 1046.2) | 199.8 (121.3, 278.3) | 2047.4 (1770.8, 2324) | 514 (454.2, 573.8) | 1256.2 (703.7, 1808.7) | 1298.8 (1031.7, 1565.9) |
| 14 | 5 | Mean (95% CI) | 6.8 (3.2, 10.4) | 3.4 (0.6, 6.2) | -23 (-136, 90) | 2615 (1616.5, 3613.5) | 876.8 (674.2, 1079.4) | 8944.6 (5480.8, 12408.4) | 274 (173, 375) | 873.6 (729.8, 1017.4) | 205.8 (130.7, 280.9) | 2184 (1468.9, 2899.1) | 506.6 (399.4, 613.8) | 1164.8 (704.7, 1624.9) | 1257.6 (814.2, 1701) |
| 21 | 5 | Mean (95% CI) | 7.6 (4.7, 10.5) | 3.3 (1.2, 5.4) | -13.4 (-87.2, 60.3) | 2559.6 (1699.4, 3419.8) | 870.8 (698, 1043.6) | 9489.2 (6788, 12190.4) | 366 (318.3, 413.7) | 899.6 (804.1, 995.1) | 189.8 (127.1, 252.5) | 1991.8 (1708.1, 2275.5) | 511 (441.5, 580.5) | 1292.4 (751.1, 1833.7) | 1334.4 (1035.4, 1633.4) |

**Supplementary Table 2.** **Expression of Immune Markers in Intermediate Monocytes in NoTD Volunteers.**  The table displays the means and 95% CI of the assessed markers at days 0, 1, 2, 4, 7, 9, 14 and 21 of the study. No differences significant differences in the expression of the markers between day 0 and other study days was identified.

**Supplementary Table 3.** **Expression of Immune Markers in Non-Classical Monocytes in NoTD Volunteers.**  The table displays the means and 95% CI of the assessed markers at days 0, 1, 2, 4, 7, 9, 14 and 21 of the study. No differences significant differences in the expression of the markers between day 0 and other study days was identified.

|  |  |  | **α4β7** | **TNF-α** | **CD163** | **HLA-DR** | **CD285** | **CD36** | **CD163** | **CD257** | ***S*. Typhi** | **CD354** | **CD284** | **CD86** | **CD274** |
| --- | --- | --- | --- | --- | --- | --- | --- | --- | --- | --- | --- | --- | --- | --- | --- |
| **Days** | **n** | **Stats Displayed** | **%** | **%** | **MFI** | **MFI** | **MFI** | **MFI** | **MFI** | **MFI** | **MFI** | **MFI** | **MFI** | **MFI** | **MFI** |
| 0 | 5 | Mean (95% CI) | 5.8 (3.6, 7.9) | 0.9 (0.5, 1.3) | -148.8 (-173.4, -124.2) | 1050.8 (849.4, 1252.2) | 545 (435.5, 654.5) | 3977.8 (2291, 5664.6) | 111.1 (41.4, 180.7) | 694.6 (617.3, 771.9) | 163.6 (113.9, 213.3) | 1172.4 (1028.9, 1315.9) | 400.6 (381.6, 419.6) | 838.6 (413.8, 1263.4) | 860.2 (701.7, 1018.7) |
| 1 | 5 | Mean (95% CI) | 5.9 (4.3, 7.4) | 1.1 (0.4, 1.9) | -160 (-197, -123) | 1119.8 (921.6, 1318) | 565.8 (432.6, 699) | 3872 (1436.8, 6307.2) | 128.2 (62.9, 193.5) | 713.4 (627.8, 799) | 173.4 (111, 235.8) | 1253.4 (1025.4, 1481.4) | 409.6 (360.4, 458.8) | 910.8 (430.3, 1391.3) | 838.4 (653, 1023.8) |
| 2 | 4 | Mean (95% CI) | 5.3 (3.2, 7.4) | 1 (0.1, 1.8) | -171.5 (-206.9, -136.1) | 1008 (884.9, 1131.1) | 526 (405.5, 646.5) | 3291.3 (165.9, 6416.6) | 150.9 (93, 208.7) | 711.3 (585.2, 837.3) | 159 (114.1, 203.9) | 1236.8 (1000.4, 1473.1) | 423.3 (364.7, 481.8) | 949.5 (417.2, 1481.8) | 812.3 (662.9, 961.6) |
| 4 | 5 | Mean (95% CI) | 5.2 (3, 7.5) | 1 (0.2, 1.8) | -163 (-189.3, -136.7) | 1012.4 (797.3, 1227.5) | 558.8 (422.2, 695.4) | 3819.4 (1418.2, 6220.6) | 134.8 (73.2, 196.5) | 715 (610.1, 819.9) | 185.8 (139.3, 232.3) | 1358.6 (1143, 1574.2) | 429.8 (391.8, 467.8) | 1076.6 (596.3, 1556.9) | 879.8 (681.7, 1077.9) |
| 7 | 3 | Mean (95% CI) | 4.7 (0.7, 8.6) | 0.8 (-0.3, 1.8) | -177.7 (-193.5, -161.8) | 1033.7 (792.8, 1274.5) | 510 (401.4, 618.6) | 3464.3 (-83.7, 7012.3) | 71.6 (35, 108.1) | 709.7 (556.6, 862.8) | 153.7 (95.5, 211.8) | 1172.3 (1011.5, 1333.2) | 403.7 (377.1, 430.2) | 1025.7 (512.9, 1538.5) | 781.3 (582, 980.7) |
| 9 | 5 | Mean (95% CI) | 5.3 (3.6, 6.9) | 0.8 (0.4, 1.2) | -158.8 (-196.9, -120.7) | 1127 (928.1, 1325.9) | 557 (439.3, 674.7) | 3618 (1356, 5880) | 117.6 (77.1, 158) | 725.8 (642.1, 809.5) | 185.6 (110.9, 260.3) | 1189.4 (1024.4, 1354.4) | 416.2 (385.5, 446.9) | 1106 (601.5, 1610.5) | 897.4 (684.6, 1110.2) |
| 14 | 5 | Mean (95% CI) | 2.9 (1.1, 4.6) | 1.2 (0.2, 2.2) | -159.2 (-180.5, -137.9) | 1091.2 (861.8, 1320.6) | 752.4 (323.7, 1181.1) | 3162.6 (554.1, 5771.1) | 97.6 (39, 156.3) | 671.4 (560.8, 782) | 195.7 (121.7, 269.7) | 1740.2 (576.7, 2903.7) | 436.4 (362.7, 510.1) | 889 (416.6, 1361.4) | 852 (563.2, 1140.8) |
| 21 | 5 | Mean (95% CI) | 5.5 (2.7, 8.3) | 0.9 (0.3, 1.5) | -157.8 (-184.7, -130.9) | 1104.6 (878.4, 1330.8) | 589.4 (479.5, 699.3) | 4614 (2019.8, 7208.2) | 119.5 (65.2, 173.9) | 703.8 (626.1, 781.5) | 186 (121.2, 250.8) | 1210.8 (1022.8, 1398.8) | 414.8 (388.6, 441) | 1159.6 (657.1, 1662.1) | 927.2 (706.7, 1147.7) |
